# Migraine and increased risk of developing open angle glaucoma: a population-based cohort study

**DOI:** 10.1186/s12886-019-1062-9

**Published:** 2019-02-13

**Authors:** Jehn-Yu Huang, Chien-Chia Su, Tsing-Hong Wang, I-Ju Tsai

**Affiliations:** 1 Department of Ophthalmology, National Taiwan University Hospital College of Medicine, National Taiwan University, No. 7, Chung-Shan S. Rd., Taipei, Taiwan; 2 National Taiwan University College of Medicine, Taipei, Taiwan; 3 Graduate Institute of Clinical Medicine, College of Medicine, National Taiwan University, Taipei, Taiwan; 4 Department of Ophthalmology, National Taiwan University Hospital, Hsin-Chu Branch, Hsin-Chu, Taiwan

**Keywords:** Age-adjusted Charlson comorbidity index, Migraine, Open-angle glaucoma

## Abstract

**Background:**

Migraine is linked to endothelial dysfunction and is considered to be a systemic vasculopathy. Interestingly, systemic vascular diseases also occur in glaucoma patients and are considered to be vascular risk factors. Whether migraine is simply a concomitant condition in glaucoma patients or a risk factor per se for glaucoma remains unknown. Thus, in the present study, we investigated the risk for open angle glaucoma (OAG) in migraineurs using a 10-year follow-up study that employed a nationwide population-based dataset in Taiwan.

**Methods:**

This retrospective matched-cohort study used data sourced from the Longitudinal Health Insurance Database 2000. We included 17,283 subjects with migraine in the study cohort and randomly selected 69,132 subjects from the database for the comparison group. Each subject in this study was individually traced for a 10-year period to identify those subjects who subsequently received a diagnosis of OAG. The age-adjusted Charlson’s comorbidity index (ACCI) score was utilized to compute the burden of comorbidity in each subject. Multivariate regression analysis was used to assess risk factors for OAG in migraineurs. Cox proportional hazards regression was performed to compare the 10-year risk of OAG between the migraineurs and the comparison cohort.

**Results:**

Migraineurs had more vascular comorbidities than the comparison cohort. The overall incidence of OAG (per 1000 person-years) was 1.29 and 1.02, respectively, for migraineurs and the comparison cohort during the 10-year follow-up period. Age, hyperlipidemia, and diabetes mellitus were three significant risk factors for OAG in migraineurs. After adjusting for patients’ age and vascular comorbidities, migraineurs were found to have a 1.68-fold (95% confidence interval [CI], 1.20–2.36) greater risk of developing OAG than the comparison cohort, in subjects with an ACCI score of 0. This association became statistically nonsignificant in subjects with ACCI scores of 1–2 or ≥ 3.

**Conclusion:**

Migraine is associated with a higher risk of OAG for patients with no comorbidity who are aged under 50 years.

## Introduction

Migraine is a chronic neurological disease, characterized by paroxysmal attacks of unilateral throbbing headache and autonomic nervous dysfunction [1]. According to a previous study, the 1-year-period prevalence of migraine in the United States (US) was 11.7% (17.1% in women and 5.6% in men), and the prevalence rate in Taiwan is 9.1% (14.4% for women and 4.5% for men) [1, 2]. The cumulative lifetime incidence of migraine was 43% in women and 18% in men in the US [3]. Migraine prevalence is believed to be lower in Asians than in Caucasians [4].

Traditionally, migraine has been considered to be a benign disorder without long-term consequences for the brain. However, recent brain imaging studies have suggested that migraine may be a risk factor for certain structural changes in the brain, such as white matter abnormalities, and infarct-like lesions [5]. Although the exact pathophysiology of migraine remains unknown, changes in brain blood vessels, hypoperfusion disorders, and microembolization have been proposed to cause neurovascular dysfunction in migraineurs [6]. Therefore, migraine is linked to endothelial dysfunction and is considered to be a systemic vasculopathy [7, 8]. Interestingly, systemic vascular diseases also occur in glaucoma patients and are considered to be vascular risk factors [9–11]. Whether migraine is simply a concomitant condition in glaucoma patients or is a risk factor per se for glaucoma remains unknown.

The association of migraine with open angle glaucoma (OAG) has been reported in two previous population-based studies. In the Beaver Dam Eye Study, there was no difference in the frequency of OAG between a migrainous and a nonmigrainous population [12]. In the Blue Mountains Eye Study, there was no significant association between typical migraine headache and OAG in all age groups. However, the odds for OAG in individuals with a history of typical migraine headache was found to be increased among 70–79-year-olds [13]. In two other studies, patients with low-tension glaucoma had a higher frequency of migraine and headache, based on either neurobehavioral testing or a headache questionnaire [14–16].

Furthermore, migraine is an intraocular pressure-independent risk factor that is significantly associated with central visual field progression in normal-tension glaucoma patients with autonomic dysfunction [17]. Glaucomatous-like visual field defects have been detected in migraineurs by using temporal modulation perimetry [18], full-threshold 24–2 visual field tests [19], and short-wavelength automated perimetry [20, 21]. However, Usui et al. reported that the prevalence of migraine in Japanese patients with low-tension glaucoma or primary OAG is not significantly different from that in healthy subjects [22].

In a previous report, Chen and colleagues utilized Taiwan’s National Health Insurance Research Database and investigated whether migraine influence the risk of OAG. In their report, the risk of POAG was not significantly higher in the migraine cohort than in the comparison cohort (adjusted hazard ratio [aHR] = 1.15, 95% confidence intervals [CIs] = 0.93–1.42). However, there was a borderline significant trend for increased risk of POAG in young patients (age ≤ 34 years) (aHR = 1.67, 95% CIs = 0.96–2.90) [23]. In one recent study, young migraineurs with no overt cardiovascular disease were found to have increased aortic stiffness and enhanced pressure wave reflection, which may represent one possible mechanism underlying the increased cardiovascular risk in migraine patients [24]. The manner by which migraine poses a risk for OAG in patients of different age groups and comorbidities may differ. To address this question, we conducted the present study using a population-based dataset. The purpose of our study was to investigate the likelihood of OAG development after diagnosis of migraine, by using the age-adjusted Charlson comorbidity index (ACCI) score. We further assessed the hazard ratio for OAG in migraineurs with different degrees of comorbidity.

## Materials and methods

### Data source

This study was based on a sub-dataset containing 1 million beneficiaries randomly selected from all insurers, from Taiwan’s National Health Insurance Research Database (NHIRD), an electronic claims database of the Taiwan National Health Insurance (NHI) program, for the period 1996–2010. The National Health Insurance program was implemented in 1995, and covers up to 99% of the 23 million-population of Taiwan. Details of the NHI have been described elsewhere [25]. The data set included comprehensive information of longitudinal person-level data, such as demographic data, details of inpatient care and ambulatory care, and details of prescriptions. The diagnostic codes were based on the International Classification of Diseases, 9th Revision, Clinical Modification (ICD-9-CM). All personal identifications were unique, but were encrypted for privacy purposes. This research protocol was approved by the Ethics Review Board of the National Taiwan University Hospital.

### Study design and patient selection

**Fig. 1:**
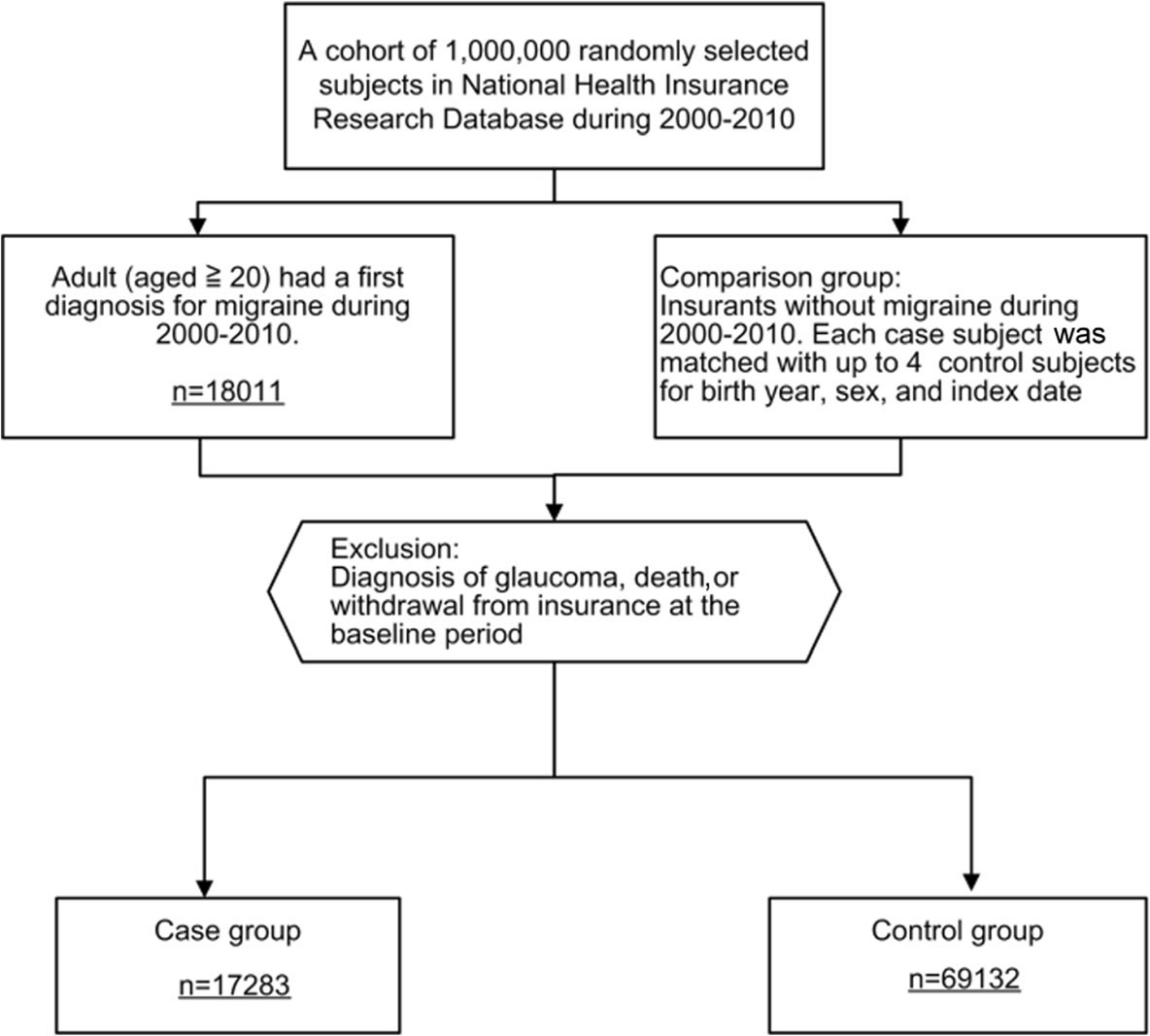
Flow diagram of the enrollment process

In this study, 1,000,000 subjects, approximately 4.3% of the population of Taiwan, were randomly selected from the NHIRD. We conducted a nationwide retrospective cohort study featuring two cohorts: a migraineur group and a control group. Figure 1 shows the subject and cohort selection. We excluded patients with a history of glaucoma diagnosis within the first year from baseline. Adults aged above 20 years who were diagnosed as having migraine (ICD-9-CM codes 346) between January 1, 2000, and December 31, 2010, and who had no history of glaucoma before enrollment, were included as the case group. To validate the diagnosis, we selected patients who had the same diagnosis code on at least three recorded visits. The first diagnostic date was defined as the index date. The age- and sex-matched control group (4 for every patient in the case group) was randomly identified from the subjects after eliminating patients who had been given a diagnosis of migraine between January 1, 2000, and December 31, 2010.

### Study variables

The comorbid medical conditions for each subject were evaluated by using the ACCI [26], which includes different chronic diseases, and a score between 1 and 6 points (1 point for myocardial infarction, congestive heart failure, peripheral vascular disease, cerebrovascular disease, dementia, chronic obstructive pulmonary disease, connective tissue disease, peptic ulcer disease, mild liver disease, and diabetes without organ damage; 2 points for diabetes with organ damage, hemiplegia or paraplegia, severe renal disease, and any malignancy, including leukemia and lymphoma; 3 points for severe liver disease; 6 points for metastatic solid tumor and HIV infection). Additional points were assigned per decade of age > 40 years. The ACCI score, the sum of the above scores, represents the measurement of the burden of medical comorbidities and is used to predict mortality. ACCI scores were further categorized into three groups (ACCI 0, 1–2, and ≥ 3). In addition to demographic characteristics and ACCI scores, we also included covariates in certain specifications; these were major disease conditions known to be associated with OAG. For all of these covariates, we required individuals to have submitted at least two claims with the same diagnosis during the study period.

### Main outcome measure

The main outcome was the diagnosis of OAG, which consisted of primary OAG and low-tension glaucoma (ICD-9-CM codes: 365.11–365.12). To validate the accuracy of diagnosis, we defined OAG if patients had three recorded visits with the same diagnosis code based on hospitalization or outpatient claims.

### Statistical analysis

The migraineurs and control patients were followed up from the index date until December 31, 2010, or death. The differences in demographic characteristics and comorbidities among the case group and control group were examined by using the chi-square test or Fisher’s exact test for categorical variables and the Wilcoxon two-sample test or Brown–Mood test for continuous variables. A Cox proportional hazards model was used to estimate the hazard ratios (HRs) and 95% confidence intervals (CIs) for the risk of developing OAG. The cumulative incidence of OAG was calculated by using the Kaplan–Meier method with the log-rank test to examine the statistical significance of the differences between the study groups. In subgroup analysis, the ACCI ≥1 group, and the combined ACCI group 1–2 and group ≥3, were compared with the ACCI = 0 group.

All statistical analyses were performed with SAS 9.3 statistical software (SAS Institute, Inc., Cary, NC, USA) while the cumulative incidence plot was drawn with R 3.0.0. A *P* value of 0.05 was considered statistically significant.

## Results

The baseline characteristics of the case and control groups after age- and sex-matching are demonstrated in Table 1. The mean age of the study cohort was 45.3 years, and 73.2% of the patients in the study were female. The matching method distributed the case and matched-controls relatively equally in terms of age and sex proportions.

**Table 1:** Demographic characteristics of patients with migraine and the control group

Variable	Patients with migraine	Control group	*p* value
	(*n* = 17,283)	(*n* = 69,132)	
Age (mean ± SD) (years)	45.3 ± 14.9	45.3 ± 14.9	0.9892
Women	12,648 (73.2)	50,592 (73.2)	1
Follow-up time (mean ± SD) (years)	6.0 ± 3.1	5.8 ± 3.2	< 0.0001
Comorbidities, person (%)			
Hyperlipidemia	1152 (6.7)	3128 (4.5)	< 0.0001
Ischemic heart disease	954 (5.5)	2179 (3.2)	< 0.0001
Hypertension	2862 (16.6)	8161 (11.8)	< 0.0001
Peripheral vascular disease	135 (0.8)	297 (0.4)	< 0.0001
Cerebrovascular disease	681 (3.9)	1460 (2.1)	< 0.0001
Dementia	65 (0.4)	226 (0.3)	0.3182
Diabetes mellitus (uncomplicated)	820 (4.7)	3341 (4.8)	0.6279
Diabetes mellitus (end-organ damage)	219 (1.3)	834 (1.2)	0.515
Age-adjusted Charlson’s score			
Score 0	9225 (53.4)	41,616 (60.2)	< 0.0001
Score 1–2	5265 (30.5)	18,365 (26.6)	
Score ≥ 3	2793 (16.2)	9151 (13.2)	

The prevalence of coexisting medical comorbidities among the patients with migraine was 16.6% for hypertension, 8.4% for chronic obstructive pulmonary disease, 6.7% for hyperlipidemia, 5.5% for ischemic heart disease, 4.7% for uncomplicated diabetes mellitus (DM), 3.9% for cerebrovascular disease, and 0.8% peripheral vascular disease. The ACCI scores were distributed as follows: 53.4% ACCI = 0, 30.5% ACCI = 1–2, and 16.2% ACCI ≥3. A significantly higher percentage of medium- and high-level comorbidity was found in patients with migraine (*P* < .0001).

**Fig. 2:**
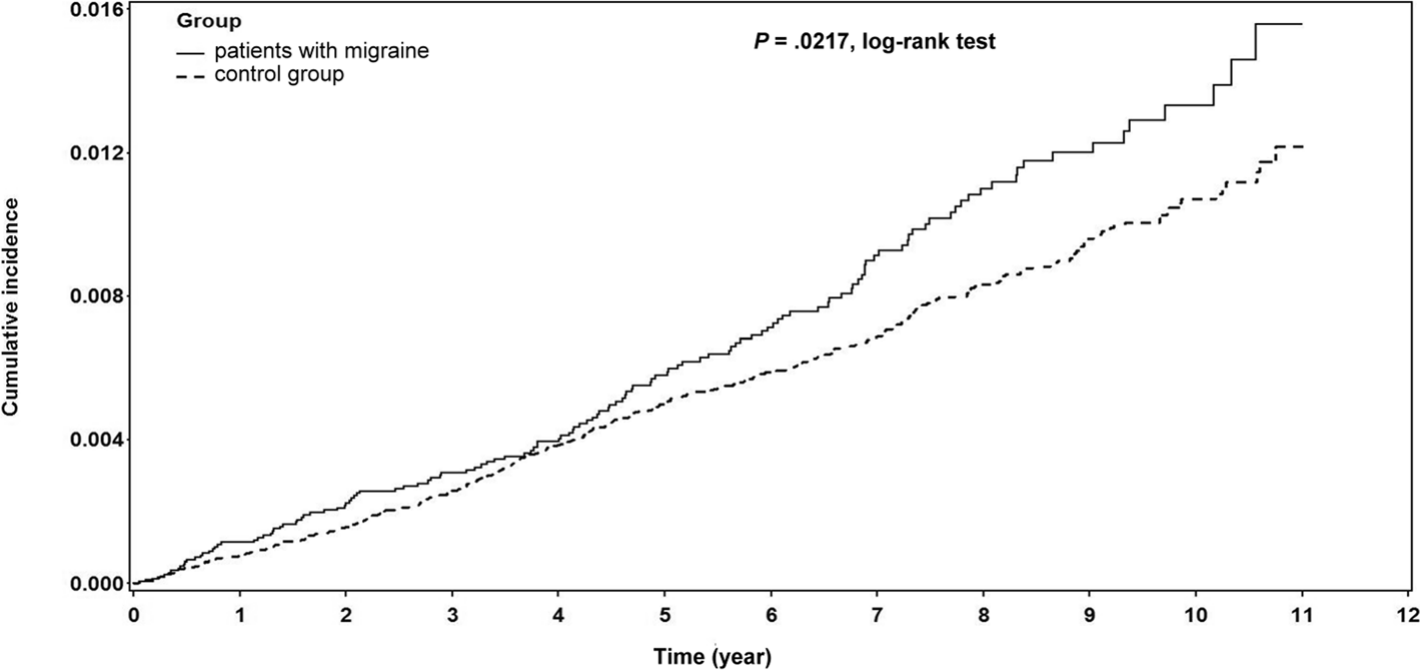
Kaplan–Meier curve of patients with migraine and of non-migrainous controls showing the development of open angle glaucoma after accounting for censoring due to death or end of observation. There was a significantly higher cumulative incidence of open angle glaucoma development in patients with migraine than in non-migrainous controls. (*P* = .0217, log-rank test)

The incidence rates per 1000 person-years of OAG among patients with migraine and among the non-migrainous controls were 1.29 and 1.02, respectively (Table 2). Figure 2 demonstrates the significant difference in the cumulative incidence rates among patients with migraine and among controls (log-rank test, *P* = .0217).

**Table 2:** Hazard ratios for open angle glaucoma (OAG) among all sampled subjects

	Patients with migraine		Control group		Compared with control			
	Event/PY	Incidence per 1000 PY	Event/PY	Incidence per 1000 PY	Crude		Adjusted	
					HR (95% CI)	*p* value	HR (95% CI)	*p* value
All patients	133/103041	1.29	413/404199	1.02	1.26 (1.04, 1.53)	0.02	1.20 (0.99, 1.47)	0.0682
Male	39/27124	1.44	122/105560	1.16	1.24 (0.87, 1.78)	0.2384	1.17 (0.81, 1.70)	0.3989
Female	94/75917	1.24	291/298640	0.97	1.27 (1.01, 1.60)	0.045	1.21 (0.96, 1.54)	0.1125
Age-adjusted Charlson’s score								
Score 0	48/55136	0.87	124/246923	0.5	1.73 (1.24, 2.42)	0.0012	1.68 (1.20, 2.36)	0.0023
Score 1–2	44/32196	1.37	172/109810	1.57	0.87 (0.63, 1.21)	0.4124	0.95 (0.68, 1.34)	0.7887
Score ≥ 3	41/15709	2.61	117/47467	2.46	1.06 (0.74, 1.51)	0.7526	1.03 (0.72, 1.47)	0.8797

Models adjusted for age, hyperlipidemia, diabetes mellitus, hypertension, ischemic heart disease, peripheral vascular disease, cerebrovascular disease

*PY* person-year, *ACCI* Age-adjusted Charlson comorbidity index, *HR* Hazard ratio

To compare the relative risks of glaucoma among the aforementioned groups, the stratified Cox regression model was used to adjust for the effects of age, sex, hyperlipidemia, ischemic heart disease, hypertension, peripheral vascular disease, cerebrovascular disease, and DM simultaneously. We further categorized patients with migraine and controls by ACCI scores as ACCI = 0, ACCI = 1–2, and ACCI ≥3. As shown in Table 2, the crude HR for all patients with migraine versus comparison group was 1.26 (95% CI, 1.04–1.53). After adjusting for the covariates, the risk of OAG was not significantly higher in the migraine group (adjusted HR = 1.20, 95% CI =0.99–1.47). The crude and adjusted HRs for patients with migraine versus the comparison group with an ACCI score of 0 were 1.73 (95% CI, 1.24–2.42) and 1.68 (95% CI, 1.20–2.36), respectively. For patients with migraine versus comparison groups with an ACCI score of 1–2 or ACCI score ≥ 3, the adjusted HRs were not significantly different (*P* = .788 and *P* = .879, respectively).

Table 3 demonstrates the results of univariate and multivariate Cox regression analyses for identifying OAG risk factors among all patients with migraine. After selecting comorbidities with a *P* value < 0.05 in univariate Cox regression, multivariate Cox regression analysis in all patients with migraine identified three OAG risk factors: age (adjusted HR = 1.03, 95% CI = 1.03–1.04), hyperlipidemia (adjusted HR =1.54, 95% CI = 1.16–2.05), and DM (adjusted HR =1.61, 95% CI = 1.23–2.11).

**Table 3:** Univariate and multivariate Cox regression analyses of glaucoma risk factors in migraine patients

	Univariate		Multivariate	
	HR (95% CI)	*p* value	HR (95% CI)	*p* value
Age	1.04 (1.03, 1.04)	< 0.0001	1.03 (1.03, 1.04)	< 0.0001
Women	0.84 (0.70, 1.02)	0.0721		
Charlson’s score				
Hyperlipidemia	2.94 (2.26, 3.81)	< 0.0001	1.54 (1.16, 2.05)	0.003
Ischemic heart disease	2.60 (1.92, 3.52)	< 0.0001	1.15 (0.83, 1.59)	0.4098
Hypertension	2.63 (2.17, 3.18)	< 0.0001	1.23 (0.98, 1.56)	0.0765
Myocardial Infarction	2.59 (0.65, 10.38)	0.1793		
Congestive Heart Failure	1.46 (0.65, 3.26)	0.3576		
Peripheral Vascular Disease	3.82 (1.98, 7.38)	0.0001	1.87 (0.96, 3.65)	0.0658
Cerebrovascular Disease	2.19 (1.47, 3.28)	0.0001	0.93 (0.61, 1.41)	0.735
Dementia	0.90 (0.13, 6.38)	0.9178		
COPD	1.66 (1.21, 2.27)	0.0017	1.02 (0.74, 1.41)	0.8841
Connective Tissue Disease	2.17 (1.19, 3.93)	0.0111	1.56 (0.86, 2.84)	0.1451
Peptic Ulcer Disease	1.57 (1.19, 2.09)	0.0015	1.05 (0.79, 1.40)	0.7382
Mild liver Disease	1.69 (1.17, 2.44)	0.0052	1.20 (0.83, 1.74)	0.3347
DM	3.19 (2.50, 4.07)	< 0.0001	1.61 (1.25, 2.24)	0.0006
Hemiplegia	1.23 (0.31, 4.92)	0.7726		
Moderate to severe Kidney Disease	3.00 (0.75, 12.01)	0.1214		
Solid Tumor	1.93 (1.14, 3.28)	0.0152	1.24 (0.73, 2.12)	0.4295

*COPD* chronic obstructive pulmonary disease, *DM* diabetes mellitus

## Discussion

In the present population-based cohort study, women constituted 73.2% of the migrainous population in this study. The prevalence of migraine in females was similar to that reported in a previous epidemiological study, which indicated that migraine is two to three times more common in women than in men [4]. We further demonstrated that the migrainous population had a higher frequency of peripheral vascular disease (*P* < .0001), cerebrovascular disease (*P* < .0001), ischemic heart disease (*P* < .0001), hypertension (*P* < .0001), and hyperlipidemia (*P* < .0001). Compared to a previous report based on the same database [23], the age and sex distributions in the migrainous and non-migrainous populations was similar. Since the present study applied stricter criteria and selected patients with the same diagnostic code on at least two, rather than one, recorded visits, the frequencies of comorbidities in both the migrainous and non-migrainous groups were less than that reported in the previous study. Nonetheless, the migrainous population had a significantly higher proportion of hypertension, hyperlipidemia, and coronary artery disease than the non-migrainous population in both our own and the previous study. Both studies demonstrated that the prevalence of DM did not differ between the migrainous and non-migrainous groups.

The above findings were similar to those of previous studies in which a higher-than-expected incidence of vascular diseases, including ischemic stroke [27], claudication, and coronary heart diseases [8, 28], have been reported in migraineurs. Patients with migraine have also been reported to have a higher frequency of cardiovascular risk factors, including hypertension and hyperlipidemia [8]. Migraine is considered to be a systemic vasculopathy and is associated with endothelial dysfunction, which is characterized by endothelial activation and impaired reactivity [7]. These vascular risk factors may be further increased by cigarette smoking and oral contraceptive use [27].

The cumulative incidence rate of OAG was 1.29 per 1000 person-years among the migrainous population. Kaplan–Meier analysis showed that the cumulative incidence of OAG was significantly higher among the migrainous population than among the control group (log-rank test, *P* = .0217). The results suggested that there is an increased risk of developing OAG in patients with migraine, as previously reported [12–16]. The present finding was also similar to those of a previous study that used the same database [23], which found that the incidence of OAG was significantly higher in the migraine cohort than in the non-migrainous cohort (log-rank test, *P* = .04).

**Fig. 3:**
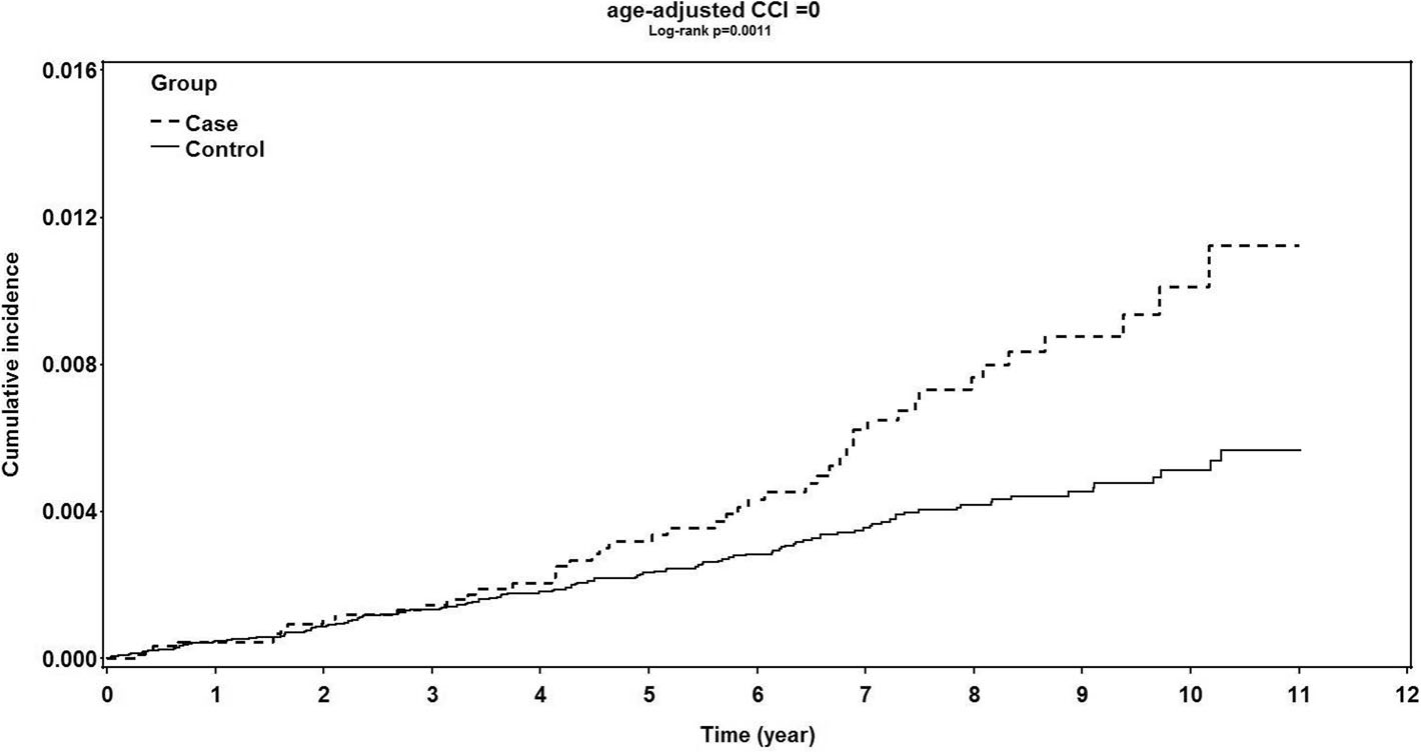
Kaplan–Meier curve of patients with migraine and of non-migrainous controls with an age-adjusted Charlson comorbidity score of 0 for development of open angle glaucoma. There was a significantly higher cumulative incidence of open angle glaucoma development in patients with migraine than in non-migrainous controls. (*P* = .0011, log-rank test)

**Fig. 4:**
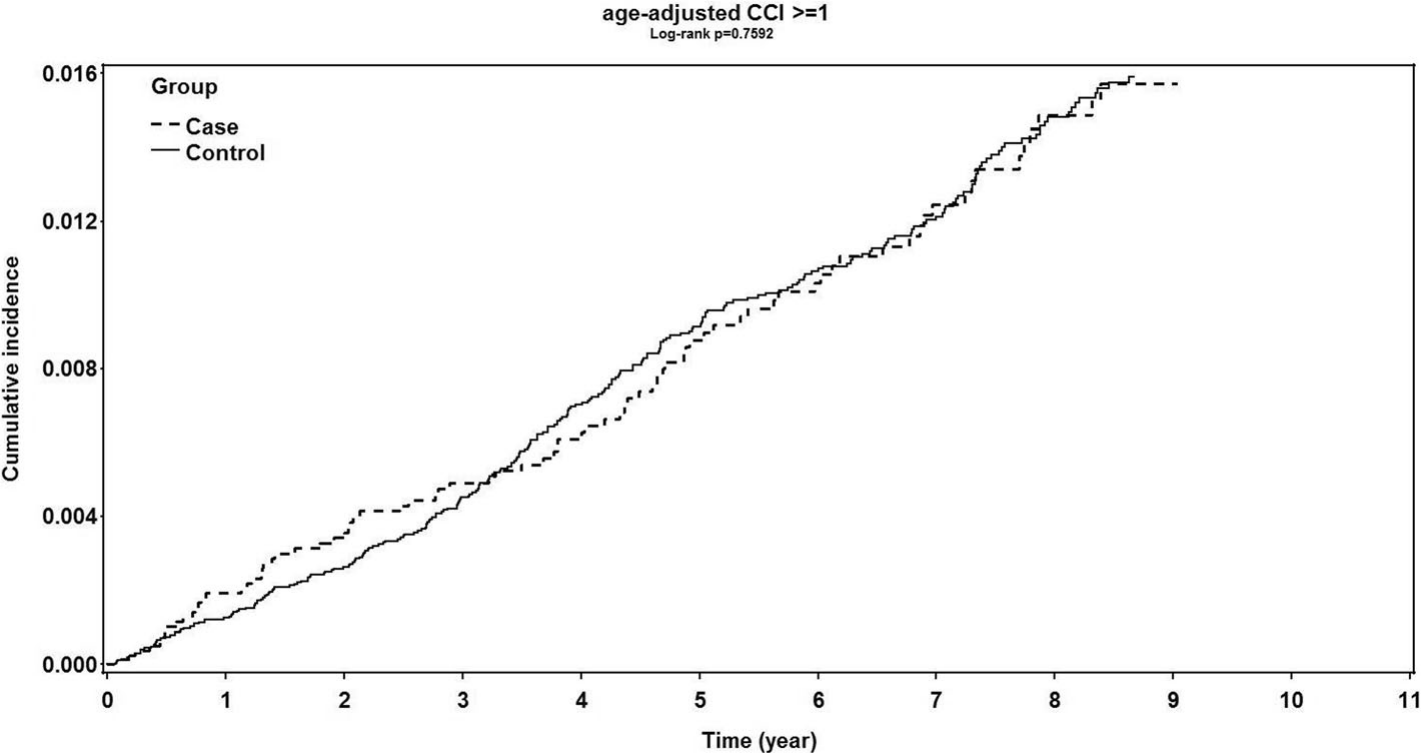
Kaplan–Meier curve of patients with migraine and of non-migrainous controls with an age-adjusted Charlson comorbidity score ≥ 1 for the development of open angle glaucoma. The cumulative incidence rate of open angle glaucoma development in patients with migraine was not significantly different from that in non-migrainous controls (*P* = .7592, log-rank test)

Due to the higher prevalence of glaucoma and higher burden of comorbidity in patients with advanced age, we used the ACCI score to quantify the disease burden of migrainous patients and non-migrainous controls in the current study. The ACCI score is a widely accepted measure of comorbidity for use with an administrative database with recorded ICD-9-CM diagnoses [29]. The CCI score, unadjusted and adjusted by age, has been proposed to be a measure of disease burden and to predict survival and outcome in previous studies [26, 30]. By using the ACCI score, we could broadly classify the subjects into categories with different disease burden levels. The cumulative incidence rates of developing OAG in patients with migraine was significantly higher than that in the non-migrainous control group among subgroups with an ACCI score of 0 (Fig. 3, log-rank test, *P* = .0011). However, the cumulative incidence of developing OAG in patients with migraine and in controls was not significantly different among the subgroups with an ACCI score ≥ 1 (Fig. 4, log-rank test, *P* = .7592).

Various studies have shown that glaucoma is associated with many risk factors, including age [31, 32], race [30], DM [33, 34], hypertension [35], and vascular disorders [10]. In the current study, we adjusted for age, hyperlipidemia, DM, hypertension, ischemic heart disease, peripheral vascular disease, and cerebrovascular disease, to analyze the risk for developing OAG in individuals with migraine. The risk of developing OAG among all migraineurs fell short of statistical significance (*P* = .0682). Subgroup analysis revealed that the risk of developing OAG was 1.68 times greater in migraineurs than in non-migrainous controls among subjects with an ACCI score of 0 (*P* = .0023). The current finding was similar to that previously reported in a study utilizing the same database: the risk of POAG was not significantly higher in patients with migraine than in non-migrainous controls after adjustment for all relevant confounding factors [23]. In contrast to the previous study, which categorized the patients into different age groups and the presence or absence of comorbidity, we evaluated patients with different ages and different levels of comorbidities simultaneously, and found that migraine was associated with a higher risk of OAG in patients without comorbidities, who were under the age of 50 years.

In the present study, comorbidities may have been under-diagnosed or not well-treated in patients with migraine in this age subgroup, as indicated by subjects with an ACCI score of 0. This finding is similar to a recent study, in which young migraineurs with no overt cardiovascular disease were found to have increased aortic stiffness and enhanced pressure wave reflection, which may represent one possible mechanism underlying the increased cardiovascular risk in migraine patients [24]. In patients with an ACCI score of 1–2 and an ACCI score ≥ 3, migraine did not increase the risk of OAG (*P* = .0788 and *P* = .8797, respectively). The effects of age and other comorbidities may mask the causal effect of migraine in these groups.

The duration of follow-up and the mortality rate were not significantly different between migraineurs and the comparison group in three subgroups with different ACCI scores. This finding may also explain the different conclusions found in previous studies.

We further used multivariate Cox regression to identify risk factors for developing OAG in a migrainous population. Age, hyperlipidemia, and DM were recognized as significant risk factors for developing OAG among the migrainous population. Migraine is postulated to represent a hyperexcitable state that sensitizes the brain to ischemic injury. The attack frequency is considered to be the relevant risk biomarker [36]. Hyperlipidemia and DM may play a role as co-occurring risk factors for the development of ischemic injury [8]. These factors may contribute to ischemic injury during glaucoma development.

This study had certain limitations. The diagnosis of migraine, OAG, and other comorbid diseases were based entirely on ICD-9-CM codes. The coding of low-tension glaucoma and OAG is less well-delineated in daily practice. The database used does not provide data on intraocular pressure, which is another important factor in glaucoma development. The severity of glaucoma and migraine were not indicated in the current database and therefore, the relationship between glaucoma progression and migraine severity could not be investigated. Furthermore, surveillance bias might also have been present, because patients with migraine might be more likely to visit a clinic than would non-migrainous individuals.

## Conclusions

Our study demonstrated the risk of developing OAG in patients with migraine in a comprehensive manner. We determined that migraineurs have a higher risk of developing OAG than do non-migrainous individuals. By classifying the burdens of risk factors in the study population, migraine was found to be significantly associated with OAG in patients without comorbidities, and who were aged less than 50 years. Further studies are needed to confirm our observations and to identify modifiable risk factors in migraineurs.

## Abbreviations

ACCI: Age-adjusted Charlson’s comorbidity index
DM: Diabetes mellitus
HR: Hazard ratio
ICD-9-CM: International Classification of Diseases, 9th Revision, Clinical Modification
NHI: National Health Insurance
OAG: Open angle glaucoma
